# Lurasidone in the treatment of schizophrenia: a 6-week, placebo-controlled study

**DOI:** 10.1007/s00213-012-2838-2

**Published:** 2012-08-19

**Authors:** Masaaki Ogasa, Tatsuya Kimura, Mitsutaka Nakamura, John Guarino

**Affiliations:** 1 Dainippon Sumitomo Pharma Co., Ltd., 6-8, Doshomachi 2-Chome, Chuo-ku, Osaka, 541-0045 Japan; 2 Kanazawa University, Kanazawa, Ishikawa Japan; 3 Setsunan University, Neyagawa, Osaka Japan; 4 Sunovion Pharmaceuticals, Inc., Fort Lee, NJ USA

**Keywords:** Lurasidone, Schizophrenia, Atypical antipsychotics, Drug therapy, Clinical trial

## Abstract

**Rationale:**

There is an unmet need in the treatment of schizophrenia for effective medications with fewer adverse effects.

**Objective:**

This study aims to evaluate the efficacy and safety of lurasidone, an atypical antipsychotic, for the treatment of schizophrenia.

**Methods:**

Patients with an acute exacerbation of schizophrenia were randomized to 6 weeks of double-blind treatment with once-daily, fixed-dose lurasidone 40 mg (*N* = 50), lurasidone 120 mg (*N* = 49), or placebo (*N* = 50). The primary efficacy measure was mean change from baseline to day 42 (last observation carried forward) in the Brief Psychiatric Rating Scale derived (BPRSd) from the Positive and Negative Syndrome Scale (PANSS).

**Results:**

Mean change in BPRSd was significantly greater in patients receiving lurasidone 40 and 120 mg/day versus placebo (−9.4 and −11.0 versus −3.8; *p* = 0.018 and 0.004, respectively). Treatment with lurasidone 120 mg/day was superior to placebo across all secondary measures, including PANSS total (*p* = 0.009), PANSS positive (*p* = 0.005), PANSS negative (*p* = 0.011), and PANSS general psychopathology (*p* = 0.023) subscales and Clinical Global Impression of Severity (CGI-S; *p* = 0.001). Treatment with lurasidone 40 mg/day was superior to placebo on the PANSS positive subscale (*p* = 0.018) and CGI-S (*p* = 0.002). The most common adverse events for patients receiving lurasidone were nausea (16.2 versus 4.0 % for placebo) and sedation (16.2 versus 10.0 % for placebo). Minimal changes in weight, cholesterol, triglyceride, and glucose levels were observed.

**Conclusions:**

In this study, which was limited by a relatively high discontinuation rate, lurasidone provided effective treatment for patients with acute exacerbation of chronic schizophrenia and had minimal effects on weight and metabolic parameters.

## Introduction

Schizophrenia is a chronic and debilitating psychiatric disorder that affects an estimated 1 % of the adult population (Regier et al. 1993). Although improvement is noted over time for some patients, the majority of patients experience at least some persisting symptoms despite treatment (Regier et al. 1993). The annual cost of schizophrenia in the USA is estimated as $63 billion with about $23 billion attributable to direct healthcare costs (Wu et al. 2005).

Although first-generation or conventional antipsychotic medications produce improvement in the positive symptoms of schizophrenia, they often result in serious adverse effects, including extrapyramidal symptoms (EPS) (Schotte et al. 1996). Conventional antipsychotic medications have been superseded by second-generation or atypical antipsychotics, which are now the mainstay of pharmacotherapy for schizophrenia (Sernyak and Rosenheck 2008). These newer agents, which are antagonists at dopamine D_2_ and 5-hydroxytryptamine 2A (5-HT_2A_) receptors, have demonstrated antipsychotic efficacy and are generally associated with a lower propensity for EPS than conventional antipsychotics (Leucht et al. 2009a; Meltzer et al. 2003; Schotte et al. 1996).

Most atypical antipsychotics display relatively high affinity for α_1_ adrenergic receptors, muscarinic receptors, and H_1_ histaminergic receptors (Ishibashi et al. 2010; Kroeze et al. 2003). The association of some atypical antipsychotics with sedation, impairment in cognitive function, and weight gain is thought to be due in part to effects at these receptors (Ishibashi et al. 2010; Kroeze et al. 2003). In addition, some atypical antipsychotics produce unfavorable changes in plasma lipid levels and glucose metabolism (American Diabetes Association et al. 2004; Henderson et al. 2005; Newcomer 2007), and there is substantial variability among atypical antipsychotics in the magnitude of weight gain and metabolic changes resulting from treatment (Newcomer 2007).

The occurrence of adverse effects can negatively affect treatment adherence. Results of a large, randomized, double-blind study sponsored by the National Institute of Mental Health showed that approximately 65 to 80 % of outpatients with chronic schizophrenia discontinue their antipsychotic medications, often because of lack of efficacy or intolerable adverse effects (Lieberman et al. 2005). The most common reasons for discontinuation because of intolerability were weight gain or metabolic effects, EPS, and sedation. These observations underscore a need for additional treatment options for patients with schizophrenia.

Lurasidone is an atypical antipsychotic that was approved by the US Food and Drug Administration (FDA) in October 2010 for the treatment of patients with schizophrenia (Latuda® 2010). In vitro receptor binding studies and preclinical behavioral studies indicate that lurasidone is an antagonist with high affinity at D_2_, 5-HT_2A_, and 5-HT_7_ receptors; moderate affinity at human α_2C_ adrenergic receptors; and weak affinity at α_2A_ adrenergic receptors (Ishibashi et al. 2010). In addition, lurasidone acts as a partial agonist at 5-HT_1A_ receptors, with moderate to high affinity. Notably, lurasidone exhibits no appreciable affinity for histamine H_1_ and muscarinic M_1_ receptors (Ishibashi et al. 2010).

We report here the findings of the first in a series of short-term, placebo-controlled studies conducted to assess the efficacy and safety of lurasidone for the treatment of schizophrenia (Nakamura et al. 2009; Meltzer et al. 2011). This 6-week, randomized, double-blind, placebo-controlled, phase 2 study evaluated the efficacy and safety of two fixed, daily doses of lurasidone (40 and 120 mg) for patients hospitalized for an acute exacerbation of schizophrenia.

## Methods

### Patients

The study enrolled men and women between 18 and 64 years of age who met *Diagnostic and Statistical Manual of Mental Disorders, 4th Edition* (DSM-IV) criteria for primary diagnosis of schizophrenia as established by the Structured Clinical Interview for DSM-IV Disorders–Clinician’s Version (SCID-CV) (First et al. 1997) and were hospitalized for an acute exacerbation of symptoms. Patients were also required to have illness duration of at least 1 year, no psychiatric hospitalization within the 3 months prior to study entry, a Brief Psychiatric Rating Scale score derived (BPRSd) from the Positive and Negative Syndrome Scale (PANSS) of ≥42 (Guy 1976; Kay et al. 1987), a score of ≥4 on two or more items of the positive symptoms subscale on the PANSS, and a Clinical Global Impression of Severity (CGI-S) score of ≥4 (moderate) (Guy 1976). Patients were excluded if they had an acute or unstable medical condition; a DSM-IV diagnosis of schizophreniform disorder, schizoaffective disorder, or the catatonic or residual types of schizophrenia; evidence of another chronic central nervous system disorder; or an existing movement disorder. Patients were also excluded if they had a history of resistance to treatment with neuroleptics, defined as failure to respond to two or more antipsychotic agents from two different classes, or clozapine, administered at adequate doses for sufficient duration, or had received depot antipsychotics within one standard treatment cycle.

All patients provided written informed consent prior to study enrollment. The study protocol and all related forms and amendments were approved by an independent ethics committee associated with each study center. The study was conducted in accordance with FDA guidance documents on Good Clinical Practice, as well as the International Conference on Harmonisation guidelines on Good Clinical Practices (1996) and the ethical principles of the 1964 Declaration of Helsinki.

### Study design and treatment

This was a 6-week, multicenter, randomized, fixed-dose, double-blind, parallel-group, placebo-controlled study conducted at 16 sites in the USA between February and December 2001. The study included three periods: a screening period of up to 14 days, a single-blind placebo washout period of up to 7 days, and a 6-week double-blind treatment period. Following the washout period, patients meeting entry criteria were randomized, in a 1:1:1 ratio, to once-daily fixed doses of lurasidone 40 mg, lurasidone 120 mg, or placebo. Patients randomized to the 120-mg/day lurasidone dose received 80 mg on day 1, and the dose was increased to 120 mg by day 6. All patients remained in the hospital during the screening and placebo washout periods and through the first 2 to 4 weeks of the double-blind treatment period. Patients with a Clinical Global Impression of Improvement (CGI-I) score of ≤4 were then eligible for discharge from the hospital and continued study participation as outpatients. Patients were discontinued from the study if they did not show sufficient improvement to permit discharge from the hospital by week 4 of the double-blind treatment period.

Treatment compliance at each study visit was calculated as the number of tablets taken (i.e., the number of tablets no longer in the package) divided by the number of tablets that should have been taken, multiplied by 100.

### Concomitant medications

Benztropine mesylate or biperiden (1–2 mg twice daily) were permitted for the treatment of EPS; prophylactic administration of medications for EPS was prohibited. Lorazepam (up to 8 mg/day), zolpidem (up to 10 mg/day), temazepam (up to 30 mg/day), or chloral hydrate (up to 1500 mg/day) were used as rescue medication for symptom exacerbation. As-needed use of these concomitant medications was permitted for no more than five consecutive days during the double-blind period until 8 hours prior to efficacy assessments. Other psychotropic medications were prohibited.

### Assessments

#### Efficacy

The primary efficacy measure was the mean change on the BPRSd score from baseline to week 6, assessed as the last observation carried forward (LOCF). The BPRSd comprises 18 items (derived from the PANSS) rated on a scale of 1 = not present to 7 = severe; therefore, the minimum possible score is 18, and the maximum possible score is 126. Secondary efficacy measures included mean change from baseline to week 6 (LOCF) on the PANSS total score, as well as PANSS positive symptoms, negative symptoms, and general psychopathology subscale scores (Lindenmayer et al. 1995); the CGI-S (Guy 1976); and the CGI-I. All efficacy assessments were completed at screening, at baseline, on days 3 and 7, and weekly thereafter (or on early termination).

#### Safety and tolerability

All adverse events volunteered or observed during the study were recorded, together with their severity and duration. Assessment of movement disorders occurred at every study visit (or early termination) and included administration of the 10-item Simpson-Angus Scale (SAS) to evaluate parkinsonism (0 = normal to 4 = most severe) (Simpson and Angus 1970), the 4-item Barnes Akathisia Scale (BAS) to evaluate akathisia (0 = normal to 5 = most severe) (Barnes 1989), and the 12-item Abnormal Involuntary Movement Scale (AIMS) to evaluate tardive dyskinesia (0 = normal to 4 = most severe) (Guy 1976). Other safety measures included 12-lead ECG, weight, vital signs, and clinical laboratory assessments (hematology, serum chemistry, and urinalysis). When possible, laboratory specimens were collected with patients in the fasted state.

### Statistical analysis

Allowing for a 10 % dropout rate prior to the first efficacy assessment, it was estimated that 44 patients would be needed per group (i.e., 132 patients total) to detect a standardized treatment difference of 0.730 between the lurasidone and placebo groups at 90 % power (two tailed) and at an alpha level of 0.050 (two sided). A 35 % dropout rate during the washout period was assumed. Therefore, it was planned to enter approximately 205 patients into the placebo washout period to ensure that 132 patients were randomized into the study.

Efficacy analyses were performed using the intent-to-treat (ITT) population, which consisted of all randomized patients who received at least one dose of study medication at the daily dose for the group (i.e., 40 mg lurasidone, 120 mg lurasidone, or placebo) and had at least one efficacy evaluation on or after day 3. Observed case analyses were also conducted for the primary and secondary efficacy measures, with completers represented by observed cases at day 42. The safety population comprised all patients who received at least one dose of study medication and was used for all safety analyses.

Comparisons among treatment groups at baseline were performed using a one-way analysis of variance, with treatment as a term in the model. For the primary efficacy measure of change from baseline to week 6 (LOCF) on the BPRSd, an analysis of covariance (ANCOVA) model was used with effects for center, treatment, and center-by-treatment interaction, and baseline BPRSd as the covariate. If the center-by-treatment interaction was not significant (*p* ≥ 0.10), it was removed from the model. If the overall ANCOVA of the baseline-to-endpoint change score was significant, pairwise comparisons were performed using a Dunnett *t* test. The type I error rate for rejecting a null hypothesis was set at 0.050 (two sided) for both the ANCOVA and the Dunnett test.

Secondary efficacy measures of change from baseline in the PANSS total and subscale scores, CGI-S, and CGI-I, were analyzed using ANCOVA models. The interaction term was excluded from the PANSS and CGI-I models.

Patients with a reduction of ≥20 % from baseline in BPRSd or with a CGI-I score of 1 or 2 were classified as treatment responders. The proportion of responders versus nonresponders was compared using a Cochran–Mantel–Haenszel test controlling for center. Cohen’s *d* treatment effect sizes were calculated for primary and secondary efficacy measures as the difference in least-squares (LS) mean change score for lurasidone (40 or 120 mg/day) and placebo (LOCF) divided by the pooled standard deviation.

For safety analyses, all statistical tests were two-sided at an alpha level of 0.05. One-way ANCOVA was used to analyze changes from baseline in the composite (total score) SAS, BAS, and AIMS scores. The Fisher exact test was used for between-group comparisons of the rates of adverse events.

## Results

**Fig. 1:**
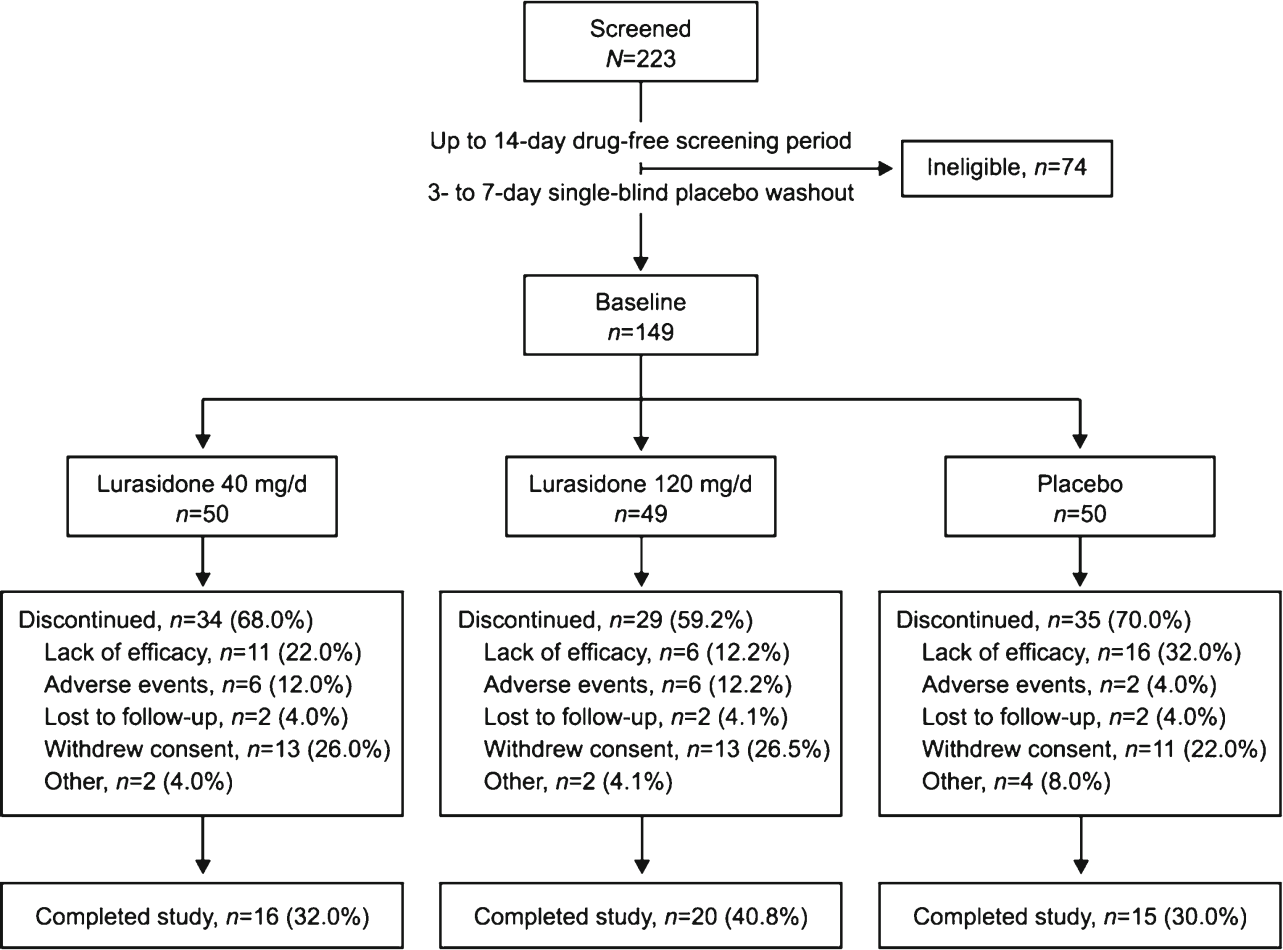
Study flow diagram

Of 223 patients who were screened, 149 met entry criteria at the end of the washout period and were randomized to double-blind treatment (50 to lurasidone 40 mg/day, 49 to lurasidone 120 mg/day, and 50 to placebo) (Fig. 1). Baseline characteristics were similar across treatment groups (Table 1). The majority of patients in each group were men who met criteria for the paranoid subtype of schizophrenia. The ITT population, used for efficacy analyses, included 49 patients assigned to lurasidone 40 mg/day, 47 assigned to lurasidone 120 mg/day, and 49 assigned to placebo. All randomized patients were included in the safety population. Overall, 51 patients completed the study: 16 (32.0 %) in the 40 mg/day group, 20 (40.8 %) in the 120-mg/day group, and 15 (30.0 %) in the placebo group. The most common reason for discontinuation was withdrawal of consent (24.8 % of randomized patients across all treatment groups) (Fig. 1). Lack of efficacy was the most common reason for discontinuation in the placebo group (32.0 %) and was cited less frequently as a reason for discontinuation in the 40 (22.0 %) and 120 mg/day lurasidone (12.2 %) groups. The placebo group had the lowest rate of discontinuation due to adverse events (4.0 %), whereas rates were higher and similar for the 40 (12.0 %) and 120 mg/day lurasidone (12.2 %) groups.

**Table 1:** Summary of patient characteristics at baseline (safety population)

Characteristic	Lurasidone (40 mg/day; *N* = 50)	Lurasidone (120 mg/day; *N* = 49)	Placebo (*N* = 50)
Sex (male; *n* (%))	36 (72.0)	36 (73.5)	42 (84.0)
Race (*n* (%))			
White	20 (40.0)	22 (44.9)	20 (40.0)
Black	25 (50.0)	24 (49.0)	25 (50.0)
Other	5 (10.0)	3 (6.1)	5 (10.0)
Age (years; mean (SD))	39.8 (9.5)	41.0 (9.0)	38.1 (9.7)
Body mass index (kg/m^2^; mean (SD))	29.5 (7.3)	29.6 (7.6)	29.4 (5.6)
Schizophrenia subtype (*n* (%))			
Paranoid	45 (90.0)	44 (89.8)	45 (90.0)
Undifferentiated	4 (8.0)	4 (8.2)	4 (8.0)
Disorganized	1 (2.0)	0 (0.0)	1 (2.0)
BPRSd			
Mean (SD)	54.6 (9.1)	52.5 (7.6)	54.4 (8.3)
Median (range)	53.0 (41.0–73.0)	52.0 (38.0–72.0)	53.5 (39.0–74.0)
PANSS total (mean (SD))	92.8 (16.1)	89.6 (13.4)	93.3 (16.4)
CGI-S (mean (SD))	4.8 (0.7)	4.7 (0.6)	4.6 (0.7)

Differences among treatment groups for sex, race, and schizophrenia subtype were assessed using a Fisher exact test; differences for age, body mass index, BPRSd, PANSS, and CGI-S were assessed using analysis of variance with treatment in the model; differences in prior medications were not evaluated statistically. All available tests were not significant (*p* > 0.25)

*BPRSd* Brief Psychiatric Rating Scale (derived from the PANSS), *CGI-S* Clinical Global Impression of Severity, *PANSS* Positive and Negative Syndrome Scale, *SD* standard deviation

### Study treatment

The mean (standard deviation) duration of exposure to study treatment was similar among patients randomized to lurasidone 40 mg/day (23.4 (15.9)  days), lurasidone 120 mg/day (23.0 (16.7)  days), or placebo (22.2 (15.8) days). Mean treatment compliance was 97.5, 99.5, and 98.9 % for the lurasidone 40 mg/day, lurasidone 120 mg/day, and placebo groups, respectively. The majority of patients received benzodiazepines at least once during the study: 90.0 % of patients in the lurasidone 40 mg/day group, 87.8 % in the lurasidone 120 mg/day group, and 82.0 % in the placebo group. The mean daily dose of lorazepam was calculated by week and ranged from 0.5 to 1.3 mg for patients receiving lurasidone 40 mg/day, 0.9 to 1.7 mg for patients receiving lurasidone 120 mg/day, and 1.2 to 2.6 mg for patients receiving placebo.

### Efficacy

**Fig. 2:**
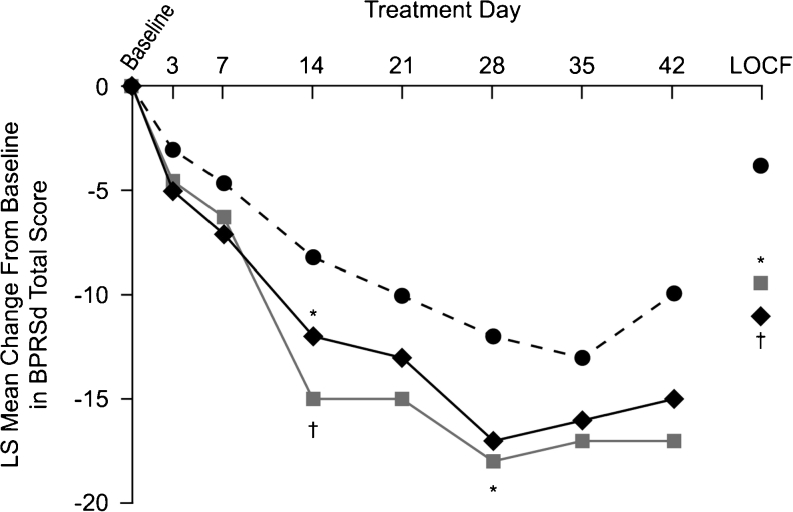
Change from baseline in BPRSd score. Least-squares (*LS*) mean change from baseline in BPRSd score. Analysis of covariance model with baseline value as covariate. Comparison with placebo based on a two-sided Dunnett *t* test and based on last observation carried forward (*LOCF*) analysis. *p < 0.05; ^†^ *p* < 0.01. *BPRSd* Brief Psychiatric Rating Scale (derived from the PANSS), *PANSS* Positive and Negative Syndrome Scale. *Circles*, placebo, *n* = 49 (LOCF), *n* = 17 (day 42); *squares*, lurasidone 40 mg, *n* = 49 (LOCF), *n* = 17 (day 42); *diamonds*, lurasidone 120 mg, *n* = 47 (LOCF), *n* = 19 (day 42)

The LS mean change in BPRSd score (primary efficacy measure) from baseline to week 6 (LOCF) was significantly greater with lurasidone 40 (−9.4; *p* = 0.018 versus placebo) and 120 mg/day (−11.0; *p* = 0.004 versus placebo) compared with placebo (−3.8) (Fig. 2). Results for completers (observed cases at day 42) are also shown in Fig. 2. Patients who completed treatment with lurasidone had numerically larger decreases in BPRSd scores than the overall LOCF analysis sample; however, there were no significant differences from placebo for either dose group for completers at day 42, perhaps as a result of small sample size.

For the PANSS total score (secondary efficacy measure), the LS mean change from baseline to week 6 (LOCF) was significantly greater in the lurasidone 120 mg/day group compared with placebo (*p* = 0.009), and a trend toward significance was noted for the lurasidone 40 mg/day group (*p* = 0.076) (Table 2). Both lurasidone doses were significantly better than placebo with regard to improvement from baseline in PANSS positive symptoms score, CGI-S, and CGI-I. Patients receiving lurasidone 120 mg/day (but not 40 mg/day) also exhibited significantly greater improvement than those receiving placebo in PANSS negative symptoms and general psychopathology scores. Compared with the overall sample, study completers showed greater improvement with lurasidone (40 or 120 mg/day) relative to placebo on each PANSS and CGI measure, but differences failed to reach statistical significance (data not shown).

**Table 2:** Change from baseline to week 6 (LOCF) in efficacy measures (intent-to-treat population)

Efficacy measure	Lurasidone (40 mg/day; *N* = 49)	Lurasidone (120 mg/day; *N* = 47)	Placebo (*N* = 49)
BPRSd			
LS mean change (SE)	−9.4 (1.6)	−11 (1.6)	−3.8 (1.6)
LS mean difference (SE)	−5.6 (2.1)	−6.7 (2.2)	
Effect size	0.53	0.65	
* p* value	0.018	0.004	
PANSS total score			
LS mean change (SE)	−14 (2.7)	−17 (2.7)	−6.2 (2.7)
LS mean difference (SE)	−7.6 (3.7)	−11 (3.7)	
Effect size	0.42	0.60	
* p* value	0.076	0.009	
PANSS positive symptoms			
LS mean change (SE)	−4.6 (0.8)	−5.1 (0.8)	−1.8 (0.8)
LS mean difference (SE)	−2.8 (1.1)	−3.3 (1.1)	
Effect size	0.53	0.63	
* p* value	0.018	0.005	
PANSS negative symptoms			
LS mean change (SE)	−2.7 (0.8)	−4.0 (0.8)	−1.0 (0.8)
LS mean difference (SE)	−1.7 (1.0)	−2.9 (1.1)	
Effect size	0.33	0.56	
* p* value	0.177	0.011	
PANSS general psychopathology			
LS mean change (SE)	−5.8 (1.5)	−7.8 (1.5)	−2.5 (1.5)
LS mean difference (SE)	−3.3 (2.0)	−5.3 (2.1)	
Effect size	0.33	0.53	
* p* value	0.185	0.023	
CGI-S			
LS mean change (SE)	−0.8 (0.2)	−0.8 (0.1)	−0.1 (0.1)
LS mean difference (SE)	−0.7 (0.2)	−0.7 (0.2)	
Effect size	0.67	0.68	
* p* value	0.002	0.001	
CGI-I			
LS mean (SE)	3.3 (0.2)	3.2 (0.2)	4.1 (0.2)
LS mean difference (SE)	−0.8 (0.3)	−0.9 (0.3)	
Effect size	0.62	0.68	
* p* value	0.006	0.002	

Analysis of covariance models based on last observation carried forward with center and treatment as effects and baseline value as covariate

Comparison with placebo was performed using a two-sided, 0.050 Dunnett *t* test

*BPRSd* Brief Psychiatric Rating Scale (derived from the PANSS), *CGI-I* Clinical Global Impression of Improvement, *CGI-S* Clinical Global Impression of Severity, *CI* confidence interval, *LS* least-squares, *PANSS* Positive and Negative Syndrome Scale, *SE* standard error

**Fig. 3:**
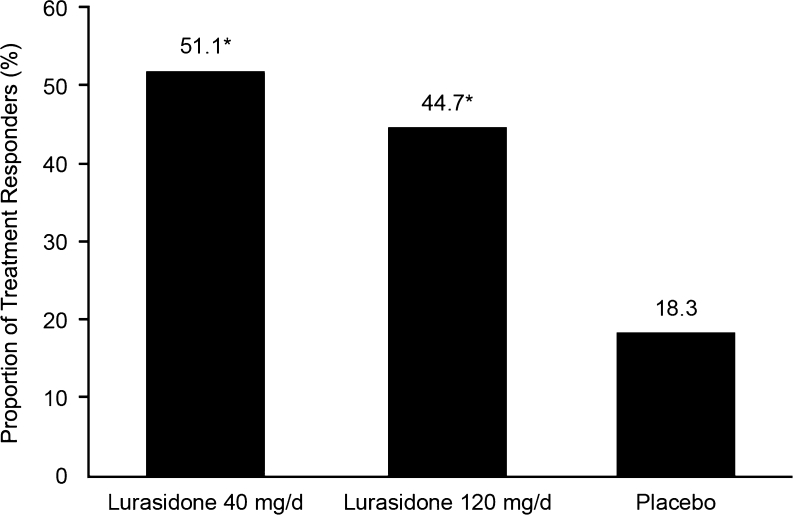
Rates of treatment response. Proportion of patients classified as treatment responders based on a reduction of ≥20 % from baseline to week 6 (LOCF) in BPRSd score. Lurasidone 40 mg/day (*n* = 49); lurasidone 120 mg/day (*n* = 47); and placebo (*n* = 49). *p < 0.01 compared with placebo. *BPRSd* Brief Psychiatric Rating Scale (derived from the PANSS), *LOCF* last observation carried forward, *PANSS* Positive and Negative Syndrome Scale

The proportion of treatment responders based on a ≥20 % decrease from baseline in BPRSd score at week 6 (LOCF) was significantly higher for the lurasidone 40 (51.0 %) and 120 mg/day (44.7 %) treatment groups compared with the placebo group (18.4 %; *p* < 0.005 for both comparisons) (Fig. 3). Similarly, a greater proportion of patients in the lurasidone 40 (36.7 %) and 120 mg/day (30.4 %) groups had a CGI-I score of 1 or 2 (very much or much improved) at week 6 (LOCF) compared with patients in the placebo group (12.2 %; *p* < 0.05 for both comparisons). The effect size (LOCF) on the BPRSd was 0.53 for lurasidone 40 mg/day and 0.65 for lurasidone 120 mg/day. Effect sizes for secondary efficacy measures are shown in Table 2.

### Tolerability and safety

#### Adverse events

The rate of adverse events occurring in ≥5 % of patients is summarized in Table 3. The most commonly reported adverse events for patients receiving lurasidone were nausea (16.2 %), sedation (16.2 %), akathisia (11.1 %), dizziness (11.1 %), and headache (11.1 %). More patients receiving lurasidone 120 mg/day reported nausea and akathisia (22.4 and 14.3 %, respectively) than those receiving lurasidone 40 mg/day (10.0 and 8.0 %, respectively). The majority of adverse events were mild to moderate in intensity. The frequency of adverse events classified as severe was similar (6.0–6.1 %) for the lurasidone and placebo groups.

**Table 3:** Treatment-emergent adverse events occurring at a rate of ≥5 % for any dose of lurasidone (safety population)

Adverse event (*n* (%))	Lurasidone (40 mg/day; *N* = 50)	Lurasidone (120 mg/day; *N* = 49)	Placebo (*N* = 50)
Any adverse event	40 (80.0)	38 (77.6)	36 (72.0)
Nausea	5 (10.0)	11 (22.4)*	2 (4.0)
Sedation	9 (18.0)	7 (14.3)	5 (10.0)
Headache NOS	8 (16.0)	3 (6.1)	5 (10.0)
Akathisia	4 (8.0)	7 (14.3)*	0 (0)
Dizziness excluding vertigo	6 (12.0)	5 (10.2)	3 (6.0)
Dyspepsia	4 (8.0)	2 (4.1)	6 (12.0)
Somnolence	4 (8.0)	5 (10.2)	2 (4.0)
Vomiting NOS	4 (8.0)	4 (8.2)	3 (6.0)
Fatigue	4 (8.0)	1 (2.0)	4 (8.0)
Tremor	3 (6.0)	4 (8.2)	0 (0)
Insomnia	3 (6.0)	4 (8.2)	0 (0)
Diarrhea	3 (6.0)	0 (0)	4 (8.0)
Constipation	4 (8.0)	0 (0)	3 (6.0)
Back pain	2 (4.0)	3 (6.1)	1 (2.0)
Extrapyramidal disorder	2 (4.0)	3 (6.1)	0 (0)
Pain in limb	3 (6.0)	1 (2.0)	1 (2.0)
Muscle cramp	1 (2.0)	3 (6.1)	0 (0)
Any adverse event reported as severe	3 (6.0)	3 (6.1)	3 (6.0)

Comparison with placebo was performed using Fisher exact test

*NOS* not otherwise specified

*p < 0.05 versus placebo

An adverse event was cited as the reason for early treatment discontinuation for 14 patients: six (12.0 %) patients receiving lurasidone 40 mg/day, six (12.2 %) patients receiving lurasidone 120 mg/day, and two (4.0 %) patients receiving placebo. The most common adverse events leading to discontinuation were worsening of schizophrenia or other psychosis (*n* = 5), movement disorder symptoms (*n* = 3), and elevations in prolactin level (*n* = 2). Serious adverse events were reported for four patients (8.0 %) receiving lurasidone 40 mg/day, two patients (4.1 %) receiving lurasidone 120 mg/day, and four patients (8.0 %) receiving placebo, all of which were exacerbations of schizophrenia or other psychosis. No patients died during the study.

#### Extrapyramidal symptoms

Change from baseline to week 6 (LOCF) on measures of EPS and use of concomitant anticholinergic medication are shown in Table 4. There were no significant differences among treatment groups at any time point for mean SAS and AIMS scores. However, there were significant differences favoring lurasidone 40 compared with 120 mg/day in mean BAS scores (change from baseline) at week 2 (lurasidone 40 mg/day, −0.3; lurasidone 120 mg/day, 1.4; and placebo, −0.3; *p* = 0.013), week 4 (lurasidone 40 mg/day, 0.0; lurasidone 120 mg/day, 1.9; and placebo, −0.6; *p* = 0.003), and week 5 (lurasidone 40 mg/day, −0.4; lurasidone 120 mg/day, 1.0; and placebo, −0.3; *p* = 0.047) but not at week 6 LOCF endpoint (*p* = 0.490). Movement disorder adverse events reported more frequently in patients receiving lurasidone 40 and 120 mg/day versus placebo, respectively, included tremor (6.0 and 8.2 versus 0.0 %), muscle cramps (2.0 and 6.1 versus 0.0 %), extrapyramidal disorder (4.0 and 6.1 versus 0.0 %), and akathisia (8.0 and 14.3 versus 0.0 %). Three patients discontinued the study because of EPS or akathisia: one patient in the lurasidone 40 mg/day group and two patients in the lurasidone 120 mg/day group. The proportion of patients receiving concomitant benztropine for the treatment of movement disorders was higher in the lurasidone groups (24.0 % with 40 mg/day and 24.5 % with 120 mg/day) than in the placebo group (18.0 %) (Table 4).

**Table 4:** Change in extrapyramidal symptom scores and use of concomitant anticholinergic medication (safety population)

	Lurasidone (40 mg/day; *N* = 50)	Lurasidone (120 mg/day; *N* = 49)	Placebo (*N* = 50)^a^
Scale (mean (SD) change)			
SAS	0.1 (1.2)	0.0 (1.1)	−0.1 (0.9)
BAS	0.3 (2.9)	0.8 (2.7)	0.1 (2.6)
AIMS	0.6 (2.8)	0.3 (2.4)	0.7 (2.6)
Use of as-needed concomitant anticholinergic medication (*n* (%))			
Benztropine^b^	12 (24.0)	12 (24.5)	9 (18.0)

Change from baseline in SAS, BAS, and AIMS was analyzed using one-way analysis of covariance

*SD* standard deviation, *SAS* Simpson–Angus Scale, *BAS* Barnes Akathisia Scale, *AIMS* Abnormal Involuntary Movement Scale

^a^One to two missing values for scales

^b^No other antiparkinson drugs were reported

#### Body weight

There were minimal changes in mean body weight in any treatment group at week 6 (LOCF) (Table 5).

**Table 5:** Change from baseline to week 6 (LOCF) in key safety parameters

Safety parameter	Lurasidone (40 mg/day; *N* = 50)	Lurasidone (120 mg/day; *N* = 49)	Placebo (*N* = 50)
Weight (kg)			
Mean (SD) baseline value	87.4 (22.0)^a^	90.2 (25.7)^b^	89.3 (20.2)^b^
Mean (SD) change	0.3 (2.2)	0.2 (2.6)	0.0 (2.9)
Total cholesterol (mg/dL)			
Mean (SD) baseline value	212 (45.8)^c^	194 (62.8)^d^	219 (38.9)^c^
Median change	−13.0	−3.0	−11.0
Triglycerides (mg/dL)			
Mean (SD) baseline value	190 (137)^c^	272 (379)^d^	275 (187)^c^
Median change	0.0	16.5	−11.0
Serum glucose (mg/dL)			
Mean (SD) baseline value	105 (42.0)^c^	113 (46.8)^d^	98.1 (19.2)^c^
Median change	0.0	−2.0	−0.5
Prolactin (ng/mL)			
Mean (SD) baseline value	9.0 (4.7)^c^	10.8 (8.0)^d^	14.8 (23.7)^c^
Median change	3.5	7.7	−1.3
QTc interval			
Mean (SD) baseline value (ms)	421.2 (20.0)	420.8 (24.5)^a^	416.8 (20.0)
Mean (SD) change (ms)	3.0 (23.6)	−3.3 (19.9)	2.2 (21.2)
Increase of >60 ms (*n* (%))	0 (0)	0 (0)	0 (0)

When possible, blood samples for evaluation of lipid, glucose, and prolactin levels were collected with patients in the fasted state

*QTc interval* QT interval corrected, *SD* standard deviation

^a^ *n* = 48

^b^ *n* = 49

^c^ *n* = 41

^d^ *n* = 40

#### Metabolic and other laboratory tests

Change in median total cholesterol from baseline to week 6 LOCF endpoint was comparable for patients treated with lurasidone (−13 mg/dL for lurasidone 40 mg/day and −3 mg/dL for lurasidone 120 mg/day) and patients in the placebo group (−11.0 mg/dL) (Table 5). Median triglyceride levels remained unchanged in the lurasidone 40 mg/day group, increased by 16.5 mg/dL from baseline to week 6 LOCF endpoint in the lurasidone 120 mg/day group, and decreased by −11 mg/dL in the placebo group (Table 5). Median serum glucose levels were either unchanged or minimally decreased from baseline to week 6 LOCF endpoint in all groups (Table 5). There were no clinically significant hematology laboratory test results or urinalysis results reported. Clinically significant markedly abnormal chemistry values were observed in one patient taking lurasidone 120 mg/day (elevated alanine aminotransferase (ALT), aspartate aminotransferase (AST), and triglycerides), and one patient in the placebo group (elevated ALT and AST). For one patient randomized to lurasidone 40 mg/day, hyperglycemia of 3+ glucosuria and elevated ALT were observed at baseline, prior to the first dose of study medication.

#### Prolactin

Median prolactin levels at week 6 LOCF endpoint were modestly increased relative to baseline in the lurasidone 40 (3.5 ng/mL) and 120 mg/day (7.7 ng/mL) groups but not in the placebo group (−1.3 ng/mL) (Table 5). A sex difference was observed, with lurasidone producing greater increases in prolactin in women than in men. Among women, the median change in prolactin at week 6 LOCF endpoint was 3.8 ng/mL in the lurasidone 40 mg/day group, 21.1 ng/mL in the lurasidone 120 mg/day group, and 0.4 ng/mL in the placebo group. Among men, smaller median changes in prolactin were observed in the lurasidone 40 (3.4 ng/mL) and 120 mg/day (5.6 ng/mL) treatment groups; the median prolactin level in the placebo group decreased by 1.4 ng/mL. Two patients (one female patient in the lurasidone 40 mg/day group and one female patient in the lurasidone 120 mg/day group) discontinued the study because of elevated prolactin based on predetermined elevations (>200 ng/mL). No prolactin-related clinical symptoms (e.g., galactorrhea) were observed.

#### Physical examination and vital signs

There were no clinically significant differences between lurasidone (40 or 120 mg/day) and placebo in changes in vital signs at any assessment.

#### Electrocardiogram

Treatment with lurasidone was not associated with any clinically significant treatment-emergent ECG abnormalities. The results of ECG were comparable at all assessments. The mean QT interval corrected (QTc) was slightly decreased (−3.3 ms) in the lurasidone 120 mg/day group and showed a small mean increase in both lurasidone 40 mg/day (3.0 ms) and placebo (2.2 ms) groups (Table 5), but changes from baseline were not clinically meaningful. No patients in any of the three treatment groups had an increase in QTc interval of >60 ms. One patient, a 56-year-old woman in the placebo group, discontinued due to nonspecific ST-T segment ECG abnormalities.

## Discussion

This was a phase 2 placebo-controlled study of lurasidone conducted in patients with schizophrenia and, as such, it included a limited sample size and was exploratory in nature. The study was not statistically powered to detect treatment effect sizes of the magnitude typically seen in placebo-controlled studies of atypical antipsychotic medications (Leucht et al. 2009b). Nonetheless, the results of this study indicate that lurasidone, at fixed daily doses of 40 and 120 mg administered for 6 weeks, was an effective treatment for patients experiencing an acute exacerbation of chronic schizophrenia, with treatment effect sizes comparable to or greater than those in other clinical studies of atypical antipsychotics (Leucht et al. 2009b). Lurasidone 40 and 120 mg/day produced significantly greater improvement than placebo on the primary efficacy measure, the change from baseline to week 6 (LOCF) in BPRSd score. On secondary efficacy measures, treatment with lurasidone 40 or 120 mg/day resulted in significantly greater improvement than placebo in PANSS positive symptoms scores, CGI-S, and CGI-I. The 120 mg/day dose also provided significant improvement in PANSS total, negative symptoms, and general psychopathology scores. The proportion of treatment responders was similar in the lurasidone 40 and 120 mg/day groups and was significantly greater than in the placebo group.

Findings from this study have been replicated and extended in a larger (*N* = 478) phase 3 study with a similar patient population (Meltzer et al. 2011). This 6-week, randomized, double-blind, placebo-controlled study also included olanzapine as an active comparator to confirm assay sensitivity. Results demonstrated that lurasidone 40 and 120 mg/day produced significantly greater improvement than placebo in PANSS total score (the primary outcome measure), PANSS subscale scores, and CGI-S score (Meltzer et al. 2011). Based on a post-hoc analysis, there were no significant differences in efficacy for lurasidone versus olanzapine (Meltzer et al. 2011).

In the present study, lurasidone 120 mg/day significantly improved PANSS total score (secondary measure) compared with placebo (*p* = 0.009), and a trend toward significance was observed for lurasidone 40 mg/day (*p* = 0.076). The ability to detect a significant effect for lurasidone 40 mg/day on PANSS total score may have been limited by the relatively small sample size in this study.

The efficacy of lurasidone in the treatment of schizophrenia was also demonstrated in a study that evaluated an intermediate dose, 80 mg/day, for the treatment of schizophrenia (Nakamura et al. 2009). In that 6-week, randomized, double-blind, placebo-controlled study (*N* = 180), patients receiving lurasidone 80 mg/day showed significantly greater improvement from baseline to week 6 (LOCF) in BPRSd (primary measure) than patients receiving placebo (Nakamura et al. 2009). Lurasidone 80 mg/day also produced significantly greater improvement than placebo on secondary measures, including the PANSS total score, PANSS subscale scores (positive, negative, cognitive, and general psychopathology subscales), and CGI-S (Nakamura et al. 2009).

Metabolic adverse effects associated with atypical antipsychotic therapy, including weight gain and changes in lipid and glucose levels, are a cause of substantial concern. Treatment with atypical antipsychotic medications has been linked to increased risk of obesity, diabetes, and dyslipidemia for patients with schizophrenia (American Diabetes Association 2004). According to a meta-analysis including head-to-head comparisons of nine atypical antipsychotic medications (amisulpride, aripiprazole, clozapine, olanzapine, quetiapine, risperidone, sertindole, ziprasidone, and zotepine), some agents were associated with considerably greater weight gain and metabolic adverse effects than others (Rummel-Kluge et al. 2010). Specifically, clozapine and olanzapine produced the greatest elevations in weight, cholesterol, and glucose. Quetiapine, risperidone, and sertindole showed intermediate elevations in these metabolic parameters. Aripiprazole, amisulpride, and ziprasidone were associated with small changes in weight and metabolic indices (Rummel-Kluge et al. 2010).

An important finding of the current study is that lurasidone had minimal effects on body weight and other metabolic parameters. Mean weight change from baseline was small and consistent with other studies of lurasidone (Meltzer et al. 2011; Nakamura et al. 2009). Other metabolic indices, including cholesterol, triglyceride, and glucose levels, either decreased or remained essentially unchanged, as has been reported in other studies of lurasidone (Meltzer et al. 2011; Nakamura et al. 2009; Potkin et al. 2011). Together, these findings suggest that the clinically significant weight gain and metabolic changes observed during short-term treatment with some atypical antipsychotics (Leucht et al. 2009a) are unlikely to occur with lurasidone. Lurasidone’s low propensity for weight gain and minimal effect on glucose and lipid parameters may be an important treatment consideration, especially in light of the increased rate of metabolic syndrome among patients with schizophrenia (Correll et al. 2010; Newcomer 2007). Lurasidone may be a particularly appropriate treatment for patients who are concerned about the potential for weight gain, dyslipidemia, and hyperglycemia during antipsychotic treatment.

Adverse events observed in the present study were generally comparable with those that have been previously reported in other studies of lurasidone (Meltzer et al. 2011; Nakamura et al. 2009). Most adverse events were rated as mild to moderate in intensity. The proportion of patients who experienced adverse events rated as severe was low and comparable for lurasidone and placebo (6.0–6.1 %).

Lurasidone appeared to be better tolerated at 40 than 120 mg/day in the present study. Nausea was more common among patients treated with lurasidone 120 mg/day compared with 40 mg/day. Although results of this study suggest that treatment with lurasidone at doses of 40 and 120 mg/day has a low propensity for causing EPS, the occurrence of akathisia appeared to be dose related. Treatment with lurasidone resulted in modest, dose-dependent increases in prolactin levels, which were more pronounced in female patients. Similar increases in prolactin were observed with lurasidone treatment in other short-term studies (Meltzer et al. 2011; Nakamura et al. 2009; Potkin et al. 2011). The mean increase in prolactin levels observed with lurasidone 120 mg/day was lower than that reported following short-term treatment with risperidone, paliperidone, or conventional antipsychotics (Bostwick et al. 2009). No galactorrhea or other clinically important prolactin-related adverse events were reported in this study.

Limitations of the current study include the short duration, small sample size, and high rate of discontinuation, which make it difficult to draw firm conclusions about the treatment effects of lurasidone. The all-cause discontinuation rate was 59 to 68 % for patients treated with lurasidone and 70 % in the placebo group. The average all-cause discontinuation rate in short-term (4- to 12-week) placebo-controlled studies of atypical antipsychotics is 48 % for active treatment and 60 % for placebo (Kemmler et al. 2005). In contrast to the present study, other studies of lurasidone had lower-than-average rates of discontinuation (Meltzer et al. 2011; Nakamura et al. 2009). All-cause discontinuation rates in a larger 6-week study of lurasidone 40 and 120 mg/day were 36 and 45 %, respectively, compared with 39 % for placebo (Meltzer et al. 2011). In the present study, the protocol required that patients must be discontinued from study participation if they had not improved enough by week 4 to permit hospital discharge, which may have contributed to the relatively high rates of discontinuation due to lack of efficacy. Since this was an early study of a novel medication, investigators may have had concerns about the efficacy and tolerability of the investigational medication, leading to higher discontinuation rates than observed in subsequent studies and increased use of rescue medications. Supporting this view, use of benzodiazepines occurred at similar high rates in the lurasidone and placebo groups.

In conclusion, this study, which was limited by a relatively high rate of discontinuation, suggests that short-term administration of lurasidone in doses of 40 or 120 mg/day was effective in the treatment of patients experiencing an acute exacerbation of chronic schizophrenia. Treatment with lurasidone had minimal effects on weight and metabolic parameters. Lurasidone may offer an effective once-daily treatment with few metabolic complications for patients with schizophrenia.
